# Some Modern Theories of Gout

**Published:** 1891-11-28

**Authors:** 


### The Practitioner's Mirror and Retrospect.

[*The Editor will be glad to receive offers of co-operation and contributions from members of the profession. All letters should be addressed to* The Editor, The Lodge, Porchester Square, London, W.]

### MEDICINE.

### SOME MODERN THEORIES OF GOUT.

Sydenham in 1683 gave an invaluable delineation of the symptoms of gout, but confesses that men would either think the subject incomprehensible or himself a person of very slender abilities, for he had been afflicted with it for thirty-four years, and yet knew so little of it. Indeed, his immoderate application to the work of writing upon it and dropsy brought on the severest fit of gout he ever had. This returned as often as he attempted to go on with the treatise he intended, so that only by the help of a friend did he write down the observations he has left us. He believed the disease sprang from a weakened concoction both of the solids and fluids, that is, from a failure of metabolism, chiefly in debilitated persons who had indulged immoderate appetites and had taken little exercise.

Until Garrod's experiments and treatise thirty years ago, we could do little more than describe the symptoms as mapped out by Sydenham, though, meanwhile, one great remedy, colchicum, had been found efficacious in cutting short the pain. Garrod showed that the blood in gout contained an excess of urates of soda and potash over what are present in health, that after recovery from acute attacks there is a great decrease in these urates, while in chronic gout there is always an excess; and again, that the excretion of urates by the kidneys in acute attacks is less than half the normal, and in long-continued chronic gout falls gradually to almost nothing.

Here, then, was a solid foundation to build on. He found, moreover, an excess of urates in cases of irregular gout before symptoms of joint disease occurred, and that lithate of soda is always present in gouty joints, and concludes that the deposit is the cause, and not the result of the inflammation of the joint. The first question then is,—What is the immediate cause of this deposition, or of the acute attack? Is there a sudden excess of uric acid manufactured, or a failure of excretion; or may we hold the middle view that some sudden change takes place in what we may call the normal excess of uric acid which these patients carry, precipitating it in the tissues? Garrod seems to hold that generally the deficient excretion of the kidneys raises the percentage till i crystalises in the joints, and when, perhaps, some over-exertion brings on an inflammatory condition. However, at times an attack of dyspepsia, or a heavy meal, may so increase the uric acid as to bring on the attack.

Cantani would seem to look upon gout as a local disease of the cartilages, while the excess of acid in the blood is secondary or accidental; but on this view it is hard to explain why an excess is not common where no gouty symptoms exist. Ebstein thinks that local accumulations take place. In the joints this causes necrosis, and necrosis crystallisation in the dead tissue, the neutral soluble compounds becoming acid ones of less solubility. Nor is there reason to suppose an increased production of urates in the system generally. Pfeiffer holds that an attack depends on an increase of the alkalinity of the blood dissolving out the uric acid deposits from the tissues, and flooding the body with the poison in an active form, while between the attacks the free uric acid either remains in the tissues, or passes out slowly in the urine uncombined and undissolved. He showed also that there is an excess of free uric acid in the urine of gouty patients, at least between the attacks. Thus he refers the acute attack to a solution of uric acid, and Ebstein to crystallisation.

Sir William Roberts has apparently proved that uric acid exists normally in the body only as a hyper-acid quadurate, which is freely excreted by the kidneys. When detained, however, by kidney failure or otherwise, it is transformed into the almost insoluble biurate. This accumulates in the blood, but at length is suddenly precipitated as crystals of biurate of sodium. The process is quicker if the percentage of quadurates is large, and depends on their taking up an additional atom of soda. He agrees with Pfeiffer that alkalisation (at least, the combination with soda) is the starting point of the process, but shows that the new compound is less, not more soluble, and that after a short time a further change occurs by the sudden precipitation of crystals. In the old distinction between the acidity of the insoluble free acid and that of the soluble quadurates, the insolubility of the still less acid biurates had been overlooked. Unfortunately the new pathology does not show how we may get rid of these biurates, or whether colchicum prevents the change into biurates or the crystallisation. Again, Roberts points out the danger of the administration of soda salts hastening on an attack of acute gout; but is this danger shared by potash, or does the latter by its diuretic action carry off the quadurate before it can be transformed into the biurate?

The practical danger from alkalies in some cases had long been observed. Fagge mentions that Bath waters were once valued for their power of "bringing out" an attack of gout. Dr. Haigh, too, who holds that uric acid may be stored up in the tissues and joints from failure of excretion (not from excess of production). and brought out into the blood by the administration of alkalies, speaks strongly of the risk of cerebral attacks following such treatment, but considers that salicylates will draw off the uric acid through the kidneys without danger.

Garrod, as we have seen, discovered that the excretion of urea by the kidneys in gout is less than normal, but it is also clear that the administration of alkalies increases it often to more than the normal. Now is this done by action on the kidney, or by increasing the production of urea, since Roberts shows that they (or at least soda) render the quadurates actually less soluble, or, lastly, have they the power under any circumstances of redissolving the crystalised biurates? Most probably their action on the kidneys is the important one. They cause excretion so rapidly that there is not time for conversion into biurates. Nor do compounds with potash and salicylate of soda, so far as we are aware, share the insolubility of biurate of soda. The second problem, then, is, What is the cause or source of the excess of the salts of uric acid in the blood of gouty persons?

The question as to the excess of free uric acid in the urine, which Pfeiffer and others have noticed in gouty subjects, is a most intricate one. We are met at once by the difficulty whether it is separated by the kidneys from its salts, or whether it exists in a free state in the blood. It exists normally in the spleen, and as Foster remarks, traces have been found in the lungs, the heart muscle, the pancreas, brain, and liver and blood, but its presence in the blood in a free state has been strongly denied. Dr. Haigh is endeavouring to prove that the increased vascular tension in such affections as migraine and the gouty kidney depends of the excess of uric acid stored up in the body, though when fixed in the tissues it may exert no influence. We do not intend to enter here into the vexed question whether uric acid, as a less oxidised product of proteid metabolism, is in any sense an antecedent of urea. They are probably common derivatives from the same source. The production of uric acid salts in excess instead of urea is a form of metabolism clearly not adapted to the present conditions of the body, and the upholders of a nervous origin of gout, such as Sir Dyce Duckworth, insist that some cause must be found for this production. Dr. Haigh escapes the difficulty by recourse to deficient elimination. If it is normally formed in the proportion of 1 to 33 of urea, and if we fail to excrete one grain a day, there is, in a few years, a large store to draw upon for gouty manifestations. Others refer the excess to disorders of the stomach or the liver. Duckworth notices the hereditary and paroxysmal character of gout, and compares the joint affections in ataxy. The effect of exhaustion in bringing on a paroxysm of gout, as in Sydenham's case, is doubtless an argument of some value on the nervous side, but it is possible that exhaustion depends on an excess of uric acid and other products of metabolism interfering with normal action. That nervous irritation or irritability is produced by gout is undoubted, so are nerve affections produced in lead poisoning. These are clearly not what the upholders of the nerve theory mean in saying that nerve irritation causes gout. They suggest that as vasomotor changes of the liver cause some forms of diabetes, so other forms of vasomotor neuroses cause an excessive production of uric acid; but, as Dr. Haigh remarks, we have no evidence that such excessive production ever exists. Or, again, a nerve change may cause a sudden combination of uric acid or loss of its solubility; but here Roberts' experiments seem to supply the necessary explanation from chemical facts alone. More complete analyses of the free and combined uric acid in the blood and urine, before, during, and after gouty attacks in the same individual, both when unmodified by the use of alkalies and other medicines, and after their administration, are still needed before we can determine the exact part played by the various tissues in the genesis of this disorder.

